# Applying Fast Scanning Method Coupled with Digital Image Processing Technology as Standard Acquisition Mode for Scanning Electron Microscopy

**DOI:** 10.1155/2020/4979431

**Published:** 2020-03-31

**Authors:** Eisaku Oho, Kazuhiko Suzuki, Sadao Yamazaki

**Affiliations:** ^1^Department of Electrical and Electronic Engineering, Faculty of Engineering, Kogakuin University, 2665-1 Nakano-machi, Hachioji, Tokyo 192-0015, Japan; ^2^Research & Development Center, Nohmi Bosai Ltd., 1-18-13, Chuo, Misato, Saitama 341-0038, Japan

## Abstract

This study proposes an efficient and fast method of scanning (e.g., television (TV) scan) coupled with digital image processing technology to replace the conventional slow-scan mode as a standard model of acquisition for general-purpose scanning electron microscopy (SEM). SEM images obtained using the proposed method had the same quality in terms of sharpness and noise as slow-scan images, and it was able to suppress the adverse effects of charging in a full-vacuum condition, which is a challenging problem in this area. Two problems needed to be solved in designing the proposed method. One was suitable compensation in image quality using the inverse filter based on characteristics of the frequency of a TV-scan image, and the other to devise an accurate technique of image integration (noise suppression), the position alignment of which is robust against noise. This involved using the image montage technique and estimating the number of images needed for the integration. The final result of our TV-scan mode was compared with the slow-scan image as well as the conventional TV-scan image.

## 1. Introduction

The general-purpose scanning electron microscope (SEM) has a variety of operating conditions (operational parameters, e.g., accelerating voltage, incident current, pressure, scanning mode, working distance, magnification, and detectors). According to the properties of individual specimens and purposes of observation, these operational parameters are determined as appropriately as possible to obtain a signal containing useful information because the magnitude of the SEM signal is generally inadequate to this end. In such circumstances, the slow-scan mode (single long-period scan) is one of the most important technical features in SEM. It has been long used as the standard mode of acquisition for scanning electron microscopy (SEM) to obtain images with a sufficient signal-to-noise ratio (SNR) and sharpness. However, in a full-vacuum condition of the specimen chamber, this scan rate is likely to promote charging in a nonconductive specimen. The effects of charging in SEM images lead to a wide range of circumstances, e.g., anomalous changes in apparent brightness, beam deflection, raster faults, and bursts of charge [1–4]. And charging effects were simulated quantitatively using Monte Carlo or other method [5, 6]. The suppression of charging effects remains a significant outstanding problem.

Methods to reduce the adverse effects of charging in SEM instruments in a full-vacuum condition may fall into one of two categories (this vacuum condition has a strong advantage in terms of image quality, including image resolution): those that use low accelerating voltage (LV) and those that use a kind of fast scan discussed here. Many uncoated problematic samples (nonconductive) are frequently observed using LV-SEM, and SEM manufacturers have drastically improved its resolution on demand.

The fast-scan mode (e.g., television (TV) scan) is popular and is traditionally utilized for observation of a nonconductive sample [7]. The stability of an image scanned at the TV rate is evidence that a particular charge distribution is stable [3] and is generally coupled with the simple image integration technique (frame averaging) to reduce noise in SEM images [8]. However, it is commonly believed that integrated SEM images obtained by the fast-scan mode are blurred. This blur is mainly related to specimen deformation (drift) as well as the adverse effects of charging. In this kind of operation, image integration with position alignment is sometimes used to reduce image degradation (blur), in some commercial SEM instruments. This is effective, but the results are still not as sharp as SEM images acquired in slow-scan mode. The reason for this additional blur is that the detector system has a significant problem with the characteristics of frequency. Hence, the blurring of SEM images obtained from the TV scan is anticipated, even when the image integration technique works ideally.

In our study, the two problems strongly related to the causes of image blur (image integration technology and characteristics of the detector) are solved by using digital image processing techniques and taking advantage of the clear merit of fast scanning. The results here show that the traditional slow-scan mode can be widely replaced with a fast-scan mode in the near future.

As another study with a similar purpose, a special raster scanning method, which is a combination of fast scan (horizontal direction) and slow scan by an unusual waveform (vertical direction), was used in a prototype SEM system [9]. This boasts the advantages of both fast- and slow-scan modes. However, the loads imposed by a scanning system and the digital image processing on the prototype instrument are large.

## 2. Adverse Effects of High-Frequency Characteristics in SEM Signal Detection System on TV-Scan Images and Compensating for Them

Compared with the slow scan, a fast scan requires a combination of sophisticated technologies. It includes several technologies on a deflection controlling system for scanning, electronic circuit technology, frequency characteristics of the secondary electron detector, and digital image processing. These applications have slowly but surely improved. However, characteristics of the frequency of SEM signal detection systems have not yet matured (in addition, faster scan modes tend to be used in several commercial SEMs). This situation, which can be usually ignored, strongly affects the results of this study. We use an inverse filter to resolve this situation. Therefore, characteristics of the frequency of SEM instruments need to be measured first.

Digital SEM signal output from a Hitachi S-3400N (general-purpose SEM, Hitachi High Technologies, Tokyo, Japan) was used in this study. TV-scan SEM digital video signals were continuously acquired using a personal computer controlled with LabVIEW (National Instruments, Austin, TX, USA). To obtain better results, the personal computer was equipped with a DVI3USB 3.0 video grabber for lossless video capture from a device with a digital visual interface output port (Epiphan Systems Inc., Ottawa, Ontario, Canada).

### 2.1. Influence of Degraded High-Frequency Characteristics at Each Scan Speed

**Figure 1:**
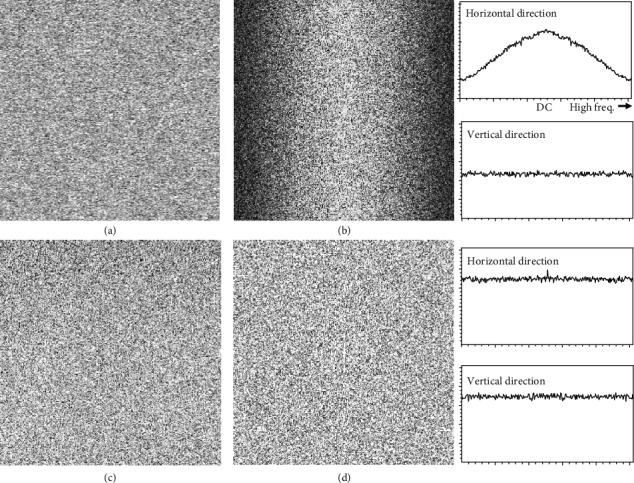
Influence of degraded high-frequency characteristics at each scan speed. (a, b) Noisy image (perfectly defocused) obtained by TV scan and its amplitude spectrum. (c, d) Noisy image (perfectly defocused) obtained by slow scan and its amplitude spectrum.

We measured characteristics of the spatial frequency of the SEM signal detection system by using noisy images (perfectly defocused) obtained by fast scan (0.04 s/image, 640 × 480 pixels; we call it “TV-scan” in this paper) and slow scan (20 s/image). The appropriate image integration technique was used on TV-scan images to adjust the amplitude of noise in them. Figures 1(a) and 1(b) show the noisy image (part of it) obtained by TV scan and the Fourier spectrum of its amplitude, respectively.  Figure 1(b) shows the estimated characteristics of the spatial frequency of the SEM instrument. A line profile (averaged values over a few hundred lines) along the horizontal (scanning) direction in Figure 1(b) shows severe degradation of the high-frequency region in question (the upper-right corner in Figure 1). Needless to say, that along the vertical direction showed no degradation. Figures 1(c) and 1(d) show the noisy images obtained by slow scan and the spectrum of its amplitude, respectively. Contrary to the results of the TV scan, both line profiles in Figure 1(d) were undegraded in this SEM condition.

### 2.2. Modifying TV-Scan Image Using Inverse Filter and Comparison of SEM Images in Terms of SNR in Each Scan Mode

**Figure 2:**
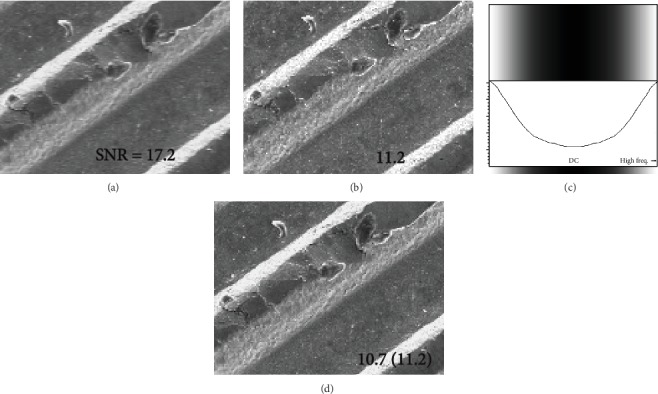
Modifying TV-scan image using inverse filter and comparison of SEM images in terms of SNR in each scan mode. (a) TV-scan image (integration of 512 images, expanded image by four times). (b) Slow-scan image. (c) Shape of inverse filter. (d) TV-scan image modified by (c).

In this scenario, we compared a TV-scan image (Figure 2(a), 15 kV; coin, integration of 512 TV-scan images) and a slow-scan image (Figure 2(b), acquired in 20 s). They were captured in nearly identical acquisition times. Because they were digitally expanded images by four times, we can easily see the differences (blur) between them. This integrated TV image was more blurred than the slow-scan image. We did not need to use an image integration technique with position alignment because Figure 2(a) was acquired at a very low magnification of 100 (we confirmed that there was no shift between images using the conventional cross-correlation function). Hence, only the degraded high-frequency characteristics of the detector system were blurred.

The same SNR value was expected from the two images (Figures 2(a) and 2(b)) when the time needed for image acquisition was the same. However, the SNR in the TV-scan image (Figure 2(a)) was considerably higher than the desired value because of the degradation described above (the characteristic of a low-pass filter shown in the horizontal line profile in Figure 1(b)). The SNR used here was equivalent to the signal standard deviation *S*_*σ*_/standard deviation in noise *N*_*σ*_, and the measured value was obtained as follows:

(1) $$\begin{array}{c} \text{SNR}=\frac{S_{\sigma }}{N_{\sigma }}=\sqrt{\frac{\text{Cov}\left(t_{1},t_{2}\right)}{\sqrt{\text{Var}\left(t_{1}\right)\cdot \text{Var}\left(t_{2}\right)}-\text{Cov}\left(t_{1},t_{2}\right)}.} \end{array}$$

This measurement formula of the SNR consists of the covariance (Cov(*t*_1_, *t*_2_)) obtained from two images (*t*_1_, *t*_2_) with an identical view and the variances (Var(*t*_1_), Var(*t*_2_)) obtained from each image [10, 11]. In this study, we used two continuously acquired TV-scan images (integrated images) to this end.

To obtain TV-scan images without degradation related to the detector system, which can show a desirable SNR value, we used the inverse filter with the shape shown in Figure 2(c). This filter was designed with reference to the line profile along the horizontal direction (characteristic of spatial frequency) in Figure 1(b). This shape is different for each SEM instrument. By multiplying the spatial frequency characteristics of the TV-scan image (Figure 2(a)) with this filter on the frequency domain, the degraded characteristics were transformed into flat characteristics, like those of the slow scan. The transformed image is shown in Figure 2(d) (modified TV-scan image). This image preserves structural details composed of one or a few pixels with an acceptable amount of image contrast in the SEM image acquired by TV scan. Additionally, the SNR value of the image shown in Figure 2(d) was similar to that of Figure 2(b) (slow scan). We think that a slightly smaller value of this SNR was obtained because of the difference in blanking periods between images acquired using the TV scan and slow scan, which is not provided here. Specifically, periods that were not directly used to generate TV-scan and slow-scan SEM images (such as the blanking period) differed between the methods. The former and the latter were roughly assumed to be 20% and 10% of the time needed for image acquisition, respectively. Then, when the SNR was reexamined by eliminating the time difference, according to our calculations, the SNR of the TV-scan images, as shown in the brackets, and those of slow-scan images were identical. Thus, the SNR values of the SEM images could be compared more accurately than before, regardless of scanning speed.

## 3. Results of Integrating TV-Scan Images and Estimating Appropriate Number of Images Used for Integration

**Figure 3:**
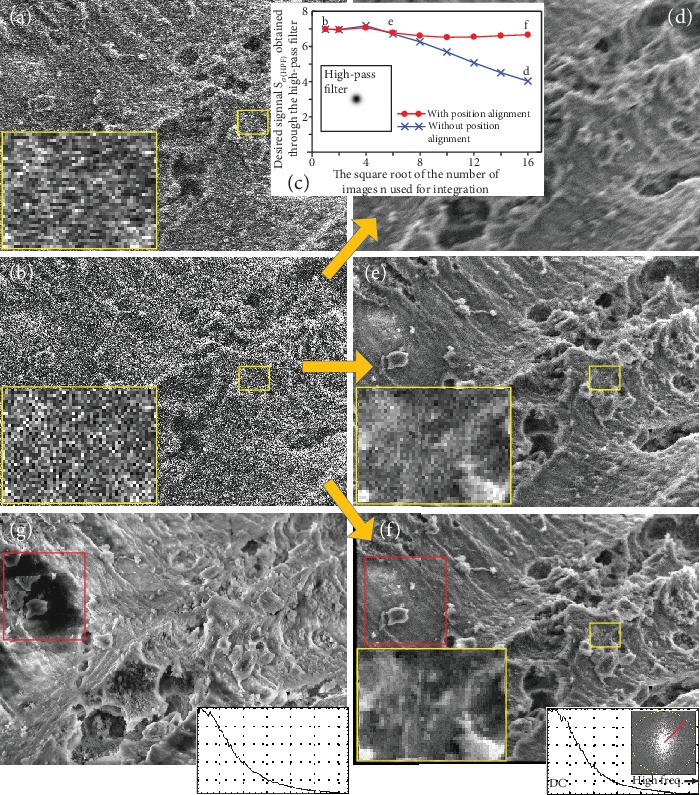
Results of integrating TV-scan images and estimating appropriate number of images used for integration. (a) TV-scan image. (b) Modified TV-scan image. (c) Graph of the measured values of the desired signal with respect to the number of images to use for image integration. (d–f) Three results obtained from different image integration conditions. (g) Slow-scan image. See text for details.

A total of 512 TV-scan images of an uncoated specimen (shell of foraminifera) were first acquired continuously (10 kV, 2000 magnification). One of these images is shown in Figure 3(a) with its expanded image (very noisy), identified by the yellow frame of the small rectangle. The difference in image quality between Figures 2(a) and 3(a) was in the magnitude of noise because the latter shows the image before integration. We then performed the abovementioned inverse filter processing to the image in Figure 3(a). Its filtered image is shown in Figure 3(b). In particular, the noise of the two expanded images had clearly different shapes. The same filter processing was applied to a series of 512 images (this filtering generated the same final results of image integration, even though it was executed at the end; this procedure was advantageous in terms of saving processing time).

The measured value of the desired signal (the square root of covariance obtained from two continuously acquired TV-scan images with an identical view, *S*_*σ*_—see the abovementioned formula) shown in Figure 3(b) is the first value of the graph in Figure 3(c) (*b* and *d*–*f* indicate the measured values obtained from Figures 3(b) and 3(d)–3(f), respectively). The signal had not yet been integrated; because of which, there was no blur. In other words, this was almost identical to the maximum value of *S*_*σ*_. To obtain a satisfactory final result nearly every time by using image integration, it was necessary to estimate the appropriate number of images to accumulate for averaging. This depended in turn on the difference in the method of image integration used, that is, whether the position alignment technique was used. In addition, it probably depended on properties of the specimen and operating conditions of the SEM. To the best of our knowledge, this estimation has not been attempted to date in our field.

To estimate the appropriate number of images to use for image integration, the desired signal *S*_*σ*_ in an integrated SEM image was measured as frequently as necessary as shown in Figure 3(c). The horizontal axis is the square root of the number of images used for integration. Three examples of image integration are shown using arrows in Figure 3. As an important step to obtain the value of *S*_*σ*_ of the integrated image, a series of the inverse filtered images were divided beforehand into odd (256 images) and even (256 images) pairs. The 256 (=16^2^) images in each group were simply integrated without position alignment first. These images, which can be regarded as two images with an identical view, were then used to obtain the values of *S*_*σ*_. One of two integrated images is shown in Figure 3(d). Because Figure 3(d) shows the simple integrated image of the uncoated specimen, we easily see image blur (image shift) caused by the charging effect. Unsurprisingly, the value *S*_*σ*_ of *d* in the graph in Figure 3(c) dropped considerably. Contrary to the result shown in Figure 3(d), when using a simple integration of 36 (=6^2^) images, the result of Figure 3(e), with its expanded image (large yellow frame), did not show blur. Of course, the value of *e* in the graph in Figure 3(c) barely degraded. This is the optimal number of images applicable to simple integration without position alignment in case of the given conditions of the SEM (a total of 72 images, 36 odd images + 36 even images), but the adverse effects of noise are still visible in the integrated image.

When using the image integration coupled with position alignment (pattern matching technique), these problems (blur and noise) were nearly perfectly solved. For this purpose, we used a form of the zero-mean normalized cross-correlation function (ZNCC), which accelerates processing speed by using the pyramid algorithm [12, 13]. The ZNCC is frequently used in pattern matching techniques and generally yields stable results, except on some difficult tasks mentioned below. As an example of image integration, Figure 3(f) shows a result using position alignment after confirming that the value of *f* (*S*_*σ*_) in Figure 3(c) did not deteriorate. We observed the structural details formed by a few pixels without disturbance due to noise in its expanded image (large yellow frame). The SNR in Figure 3(f) was close to √*n* (the number of images *n* used for integration) times that in Figure 3(b) because there was no degradation in the desired signal *S*_*σ*_. However, the image shown in Figure 3(f) is an integrated image composed of 512 images (all of 256 odd images and 256 even images used to obtain the value of *f* (*S*_*σ*_) in Figure 3(c)) for reasonable comparison with the slow-scan image shown in Figure 3(g) (identical view; acquisition time, 20 s). As mentioned above, the slow-scan images were frequently disturbed by the adverse effects of charging. In this case as well, compared with the stabilized area represented by the red frame in Figure 3(f), that of Figure 3(g) suffered from all kinds of heavy disturbances due to charging.

This position alignment method was used because the results of stable integration of the TV-scan images were always as expected. Because the SNR of the image in Figure 3(b) was 0.25, which is very low, this suggests that technique for image integration used here has position alignment function that is highly robust to noise. In addition, because it was fast on a variety of SEM images that did not have complex distortions, it is superior to state-of-the-art methods, as explained later. The processing time (i7-7Y75 CPU, 16 GB RAM) needed to obtain the image in Figure 3(f) (640 × 480 pixels) was only 10–20 seconds and depended on the area used for position alignment, i.e., the areas of the inspection image and the template image.

We compared the amplitude spectra (line profiles) of Figures 3(f) and 3(g) to confirm the performance of the proposed method in terms of image integration. They show line profiles of the normalized integrated intensities (for noise reduction) around concentric circles as a function of distance from the center of the amplitude spectrum (see the red line on the spectrum in Figure 3(f)) [14]. When comparing the shapes of line profiles, nearly no difference was found in relation to the characteristics of frequency between the slow-scan image and the integrated TV-scan image. This indicates the adequate performance of the proposed integration method (we remain worried that the line profile in Figure 3(g) was slightly altered by charging effects).

Note that this was not a simple *S*_*σ*_ but *S*_*σ*(HPF)_ obtained through a high-pass filter (HPF, spatial frequency domain) as shown in Figure 3(c). This process was performed in order to emphasize the difference in image sharpness between when the position alignment was performed and when it was not performed (high-pass filtered images are not indicated). In our case of the HPF used in Figure 3 (640 × 480 pixels), it is designed to filter large structures down to 8 pixels [15]. Its filter characteristics will need to be determined by trial and error, under each image acquisition condition, e.g., the number of pixels (when filtering a SEM image with 1280 × 960 pixels, set the parameter to 16 pixels). However, judging from the reason using this high-pass filter, we believe it is not necessary to design it strictly.

## 4. Applying Image Integration Technology to a Series of TV-Scan Images with Large Visual Field Drift

**Figure 4:**
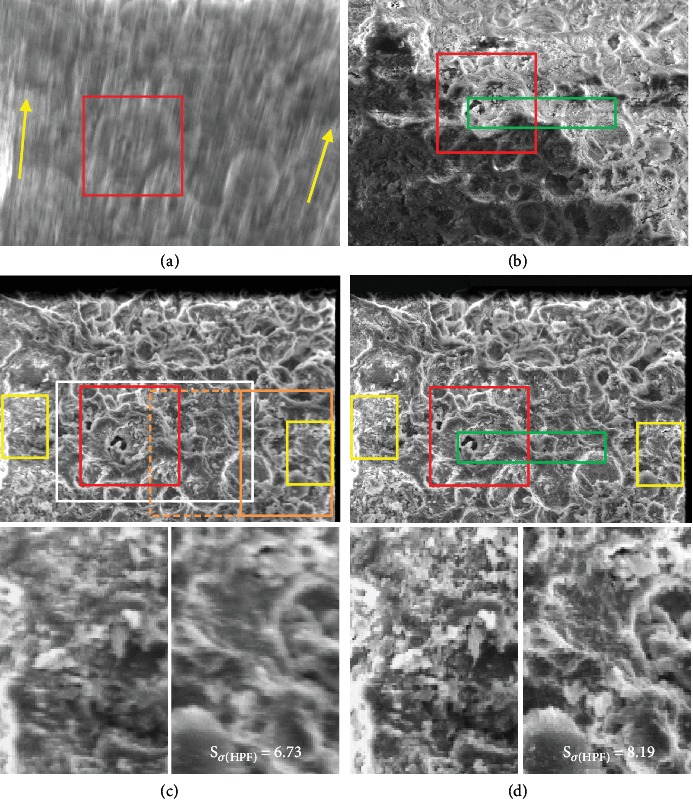
Applying image integration technology to a series of TV-scan images with large visual field drift. (a) Integrated image without position alignment. (b) Slow-scan image. (c) Integrated image with position alignment. (d) Final result of image integration using an image montage technique.

Owing to adverse effects of charging and so on, we sometimes encounter a series of TV-scan images where the field of view shifts rapidly. It may be usually difficult to perform image integration for them.  Figure 4(a) shows an integrated image without position alignment of 512 TV-scan images. From this result (significant lack of sharpness), we can understand the existence of large visual field drift in TV-scan images. In Figure 4(a), we use the same SEM operating condition to Figure 3(d), but another shell of foraminifera is adopted for intentionally receiving more severe adverse effects of charging. In the case of slow scan, those effects produce anomalous changes in apparent brightness and contrast as well as distortion of surface structures, as shown in Figure 4(b). Red frames in Figures 4(a)–4(d) indicate the same area. The adverse effects of charging in Figure 4(b) taken by slow scan will be additionally mentioned later.

Compared with Figure 4(a), the result of successful position alignment is shown in Figure 4(c). Here, an ROI (region of interest) used for position alignment, which is identified by the white frame in Figure 4(c), was widely set for the center of the image. The black area along the edges of the image occurred owing to a lack of data for alignment. This situation can be improved by suitably adjusting certain values in the image integration (an improved result is not indicated) [9], but another problem needs to be solved. In Figure 4(c), slight blurring can be observed at both ends of the integrated image (the center of the image is perfectly sharp). These positions are identified by the yellow frames. This situation is due to differences of image drift in each area (see directions of yellow arrows in Figure 4(a)). Slight but serious blurring is clear in the expanded images shown in the lower part of Figure 4(c) and occurs because the method used in this study handled only simple drifts in the visual field (translations). In case of complex distortion as in Figure 4(c), it was difficult to achieve perfect position alignment.

To solve this problem, we select three ROIs that match the yellow frames and the white frame in Figure 4(c), which have different degrees of blur, respectively. And we obtain three integrated images by using each ROI. For all three integrated images, the sharpness in the vicinity of the ROI should be very high. Finally, we used an image montage technique with a function for visible seam suppression [16] to obtain a fully combined and integrated image, as shown in Figure 4(d) and expanded images (combining three sharp partial images). The quality of the images was as expected. We can see structural details composed of one or a few pixels in these images. One reason for the adequate image quality is that no image interpolation technology is used in our method, and new pixels, which generally cause image blur, are not created. Incidentally, the blurred area at the top of the images (Figures 4(b)–4(d)) occurred owing to a shallowness of the depth of focus.

Note that the ROIs were comparatively easily determined by trial and error from the information on image sharpness provided in Figure 4(c). In order to reasonably select the ROIs, the desired signal *S*_*σ*(HPF)_ used in Figure 3(c) is helpful. Taking the ROI selection on the right end of Figure 4(c) as an example, when the yellow frame is used as the ROI, the measured value of *S*_*σ*(HPF)_ in the yellow frame is 8.19 (the maximum value). Of course, *S*_*σ*(HPF)_ of the right expanded image in Figure 4(d) is 8.19. Next, the result in the yellow frame when using an ROI (orange frame in Figure 4(c)) of twice the height and the width of the original ROI is 8.13. It is fairly difficult to visually judge the difference in image sharpness between them. Also, the result in the yellow frame when using a larger ROI (dotted orange frame, 4 times wider than the yellow frame) is 7.75. This is because there are areas with different degrees of blur in the large ROI. Of course, we can easily understand the degradation in sharpness visually. In this way, we can find the proper ROI for position alignment. For comparison, *S*_*σ*(HPF)_ in Figure 4(c) is 6.73 (it should be noted that not only the mistake in ROI selection but also the failure of position alignment by the ZNCC might reduce the measured value of *S*_*σ*(HPF)_ in some conditions).

On the contrary, to handle more severely distorted images, many studies on image registration, which is the process of estimating an optimal transformation between or among images (including techniques for detecting feature points and finding corresponding pairs), have been used in other fields [17–20]. However, it is not necessary for the image data processed in this study. Most recent research on image registration has focused on the use of deep learning for feature extraction [21, 22], although the processing speed of these functions remains low at present. In the case of the integration of SEM images, where unusual variations are expected, this type of method may be helpful and attractive. In the near future, we may use such methods as needed.

**Figure 5:**
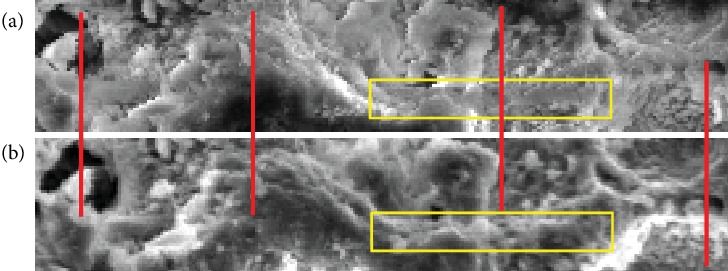
Confirmation of adverse effects of charging in slow-scan mode. (a) Expanded slow-scan image of Figure 4(b). (b) Expanded TV-scan image of Figure 4(d) (integrated image with position alignment). See text for details.

Returning to the discussion on the adverse effects of charging in the slow-scan mode, although many distortions in the SEM image of a nonconductive specimen are observed locally (influence of image or beam drift), these distortions can sometimes be inconspicuous. In abovementioned Figure 4(b), only anomalous changes in apparent brightness (a sort of the charging effects) were noticeable. Actually, this image was acquired when the adverse effects of charging had just somewhat subsided; severe effects except for anomalous changes in apparent brightness were not noticeable seemingly. However, when observing an expanded image (Figure 5(a)) identified by the green rectangle in Figure 4(b), we can find the abovementioned adverse effects caused by the slow scan. For reasonable comparison with Figure 5(a), an expanded image of Figure 4(d) taken by the proposed TV-scan mode is shown in Figure 5(b). In Figure 5(a) (slow scan), the disappearance of fine surface structures is observed in various areas (see yellow frames). In addition, when comparing the surface structures near the four red bars across the two images, distortions in the slow-scan image are clearly recognized. Specifically, it can be seen that the structures included in the left half area are relatively shifted caused by many local distortions spread throughout the slow-scan image. In contrast to this situation, we believe surface structures in Figure 5(b) (integrated image with position alignment) are more correctly produced, because there are no differences in main surface structures between the first and last image in a series of 512 TV-scan images (these images are not indicated). This is one of the most important abilities for scientific instruments.

## 5. Conclusions

This study showed that a fast scanning method coupled with a digital image processing technology applicable to a full-vacuum condition is useful for acquiring SEM images of nonconductive specimens. This fast-scan mode has the notable advantage of yielding the same quality as the original, in terms of sharpness and suppression of noise, obtained using the slow-scan mode. To realize this advantage, an inverse filter was designed and implemented based on the characteristics of the TV-scan system, and a sophisticated combination of several image processing technologies was employed. This method is especially useful for the integration of a series of sharp and noisy TV-scan SEM images acquired to cover a variety of conditions encountered when using the relevant instruments. In future work, we plan to replace the traditional slow-scan mode with a powerful fast-scan mode based on the results of this study.

## Data Availability

The data used to support the findings of this study are available from the corresponding author upon reasonable request.
